# Generating evidence for health policy in challenging settings: lessons learned from four prevention of mother-to-child transmission of HIV implementation research studies in Nigeria

**DOI:** 10.1186/s12961-018-0309-x

**Published:** 2018-04-17

**Authors:** Nadia A. Sam-Agudu, Muktar H. Aliyu, Olusegun A. Adeyemi, Frank Oronsaye, Bolanle Oyeledun, Amaka G. Ogidi, Echezona E. Ezeanolue

**Affiliations:** 1 International Research Center of Excellence, Institute of Human Virology Nigeria, Abuja, Nigeria; 2 Division of Epidemiology and Prevention, Institute of Human Virology, University of Maryland School of Medicine, Baltimore, MD United States of America; 3 Department of Health Policy, Vanderbilt University Medical Center, Nashville, TN United States of America; 4 Vanderbilt Institute for Global Health, Nashville, TN United States of America; 5 Department of Epidemiology and Public Health, University of Maryland School of Medicine, Baltimore, MD United States of America; 6 Center for Integrated Health Programs, Abuja, Federal Capital Territory Nigeria; 7 Research Resource Centre, University of Nigeria Nsukka, Enugu, Nigeria; 8 HealthySunrise Foundation, Las Vegas, NV United States of America; 9 Department of Paediatrics and Child Health, University of Nigeria Nsukka, Enugu, Nigeria

**Keywords:** HIV, PMTCT, Implementation research, Implementation science, Health systems research, Nigeria

## Abstract

**Background:**

Implementation research (IR) facilitates health systems strengthening and optimal patient outcomes by generating evidence for scale-up of efficacious strategies in context. Thus, difficulties in generating IR evidence, particularly in limited-resource settings with wide disease prevention and treatment gaps, need to be anticipated and addressed. Nigeria is a priority country for the prevention of mother-to-child transmission of HIV (PMTCT). This paper analyses the experiences of four PMTCT IR studies in Nigeria, and proffers solutions to major challenges encountered during implementation.

**Studies included and findings:**

Multicentre PMTCT IR studies conducted in Nigeria during the Global Plan’s assessment period (2011 to 2015) were included. Four studies were identified, namely The Baby Shower Trial, Optimizing PMTCT, MoMent and *Lafiyan Jikin Mata*. Major common challenges encountered were categorised as ‘External’ (beyond the control of study teams) and ‘Internal’ (amenable to rectification by study teams).

External challenges included healthcare worker strikes and turnover, acts and threats of ethnic and political violence and terrorism, and multiplicity of required local ethical reviews. Internal challenges included limited research capacity among study staff, research staff turnover and travel restrictions hindering study site visits. Deliberate research capacity-building was provided to study staff through multiple opportunities before and during study implementation. Post-study employment opportunities and pathways for further research career-building are suggested as incentives for study staff retention. Engagement of study community-resident personnel minimised research staff turnover in violence-prone areas.

**Conclusions:**

The IR environment in Nigeria is extremely diverse and challenging, yet, with local experience and anticipatory planning, innovative solutions can be implemented to modulate internal challenges. Issues still remain with healthcare worker strikes and often unpredictable insecurity. There is a dire need for cooperation between institutional review boards across Nigeria in order to minimise the multiplicity of reviews for multicentre studies. External challenges need to be addressed by high-level stakeholders, given Nigeria’s crucial regional and global position in the fight against the HIV epidemic.

## Background

The Global Plan towards the elimination of new HIV infections among children by 2015 and keeping their mothers alive (‘Global Plan’) prioritised 21 African countries for the reduction of new child HIV infections by 90% between 2011 and 2015 [1]. Key to the achievement of these goals is a sequential cascade of multiple maternal and infant services that together constitute a comprehensive, maximally effective prevention of mother-to-child transmission of HIV (PMTCT) programme [2]. Another global HIV initiative, the UNAIDS’ 90–90–90 programme, aims to achieve specific targets by 2020, namely that 90% of all people living with HIV will know their HIV status, that 90% of all those diagnosed with HIV will receive sustained antiretroviral therapy (ART), and that 90% of all people receiving ART will have viral suppression [3]. The 90–90–90 initiative seeks to end the HIV epidemic by “*bringing HIV treatment to all who need it*” [3].

Nigeria has some of the widest gaps and lowest achievements among the 21 Global Plan priority countries. In the Global Plan’s 2011 to 2015 performance period, Nigeria was able to reduce new child infections by only 21% (52,000 to 41,000), and dropped its final vertical transmission rate by only 8% (31% to 23%) [4–6]. Additionally, early infant diagnosis uptake by 2 months of age only marginally increased, from approximately 3% [7] to 9% [5]. Finally, ART coverage among pregnant Nigerian women made a modest improvement from 13% in 2009 [6] to 30% in 2015 [5]. These persistently sub-target achievements are complicated by multiple factors, including the country’s large population [8], sizeable geographic expanse [9] and the highly ethnically diverse population [10].

Nigeria is in dire need of public health strategies amenable to local adaptation, contextual application and scale-up to improve the country’s PMTCT performance. Implementation research (IR) is defined as *“the scientific study of methods to promote the systematic uptake of clinical research findings and other evidence-based practices into routine practice, and hence to improve the quality (effectiveness, reliability, safety, appropriateness, equity, efficiency) of health care*” [11]. IR is therefore a valuable tool for generating and applying evidence towards health system improvements. The per-capita quantity and quality of IR and other PMTCT/HIV research evidence generated and applied in Nigeria – and in the West and Central African region for that matter – have been low compared to other African countries and regions [12–14]. Reasons for this sub-optimal research environment include poor local research funding, infrastructure and capacity, poor integration of services, and a lack of mechanisms for putting research into practice [12, 15]. Given Nigeria’s persistent challenges with its PMTCT programme, it is especially important for quality, locally relevant IR evidence to be generated to advance the PMTCT agenda.

The objective of this paper is to consolidate and present in-country IR experiences for PMTCT IR studies conducted during the Global Plan performance period in different regions of Nigeria. In addition to being relevant to Global Plan goals, each IR study directly or indirectly addressed one or more of the UNAIDS’ 90–90–90 targets as a primary or secondary objective. The consolidated data presented here will provide insight into the most common and pervasive challenges encountered by each or all profiled studies. Ultimately, the paper aims to improve the understanding of the difficult research environment in which the local evidence most needed to facilitate Nigeria’s PMTCT agenda has to be generated.

## Methods

### Profiles of studies included

Multicentre PMTCT IR studies, initiated and/or completed in Nigeria at any time during the Global Plan’s 2011 to 2015 period, conveniently defined as January 1, 2011, to December 31, 2015, and with patient follow-up and data analysis for primary outcomes completed by December 31, 2016, were included in the review.

Studies that did not meet all three of the selection criteria were excluded. Searches were conducted within the United States National Institutes of Health’s (NIH’s) Research Portfolio Online Reporting Tool (RePORTER) and online clinical trials registries (Clinicaltrials.gov, WHO’s International Clinical Trials platform and the Pan-African Clinical Trials Registry) as well as through the Nigeria Implementation Science Alliance (NISA) network. NISA is a collaborative alliance between local implementing partners, researchers, academia and policy-makers in Nigeria, and is committed to identifying, evaluating and disseminating implementation science evidence in-country [15]. Finally, we explored relevant online bibliographic databases and websites (PubMED, Ovid MEDLINE, Web of Science) for pertinent articles published in English language that met the criteria for the following MeSH search terms: “PMTCT”, “HIV/AIDS”, “implementation science”, “implementation research”, “Nigeria”, and “research”. Various combinations of the terms were also employed.

Four distinct studies were identified for inclusion in this joint report; they were all multisite prospective IR studies focusing on PMTCT in Nigeria during the Global Plan performance period. The Baby Shower Trial and the Optimizing PMTCT study were funded by the United States’ NIH [16–19]; MoMent [20–22] and the *Lafiyan Jikin Mata* (Hausa language for ‘Excellent health for mothers’) (LJM) study [23, 24] were funded by WHO through the Integrating and Scaling up PMTCT through Implementation Research (INSPIRE) grant from Global Affairs Canada [25, 26].

After consenting to contributing to this paper, Principal Investigators for all four studies were sent a survey document by email. The survey document included a section for completing study profiles and posed open-ended questions about study experiences and internal and external challenges and solutions. Additional comments and experiences not covered by the survey were also invited. Principal Investigators ultimately completed these surveys in collaboration with their respective project coordinators where applicable/available. Data collected from each study were collated and organised into the manuscript according to the internal and external challenge/solution themes. Where available, previous publications served as additional sources of data.

### Geographical settings of studies included

**Fig. 1:**
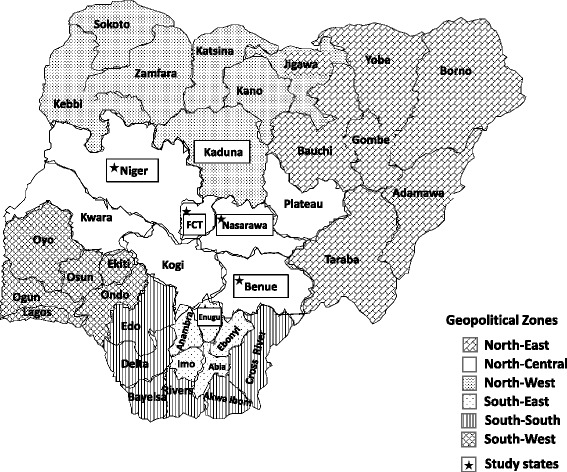
Geographical scope of the four PMTCT implementation research studies in Nigeria

All four studies were conducted exclusively in Nigeria across five states and the Federal Capital Territory (FCT), spanning three of the country’s six geopolitical zones (Fig. 1). Study settings involved both urban and rural communities; the Baby Shower and LJM trials involved a mix of urban and rural study sites, whereas MoMent and the Optimizing PMTCT studies were largely rural. In 2014, HIV prevalence among pregnant women in all six study states/territories were as follows: Benue 15.4% (highest in Nigeria), Enugu 4.9%, FCT 5.8%, Kaduna 2.2%, Nasarawa 6.3% and Niger 1.7% [27]. Although contiguously located in the North-Central zone, major ethnic groups and languages differ fairly widely in Benue (Tiv and Idoma) [28], FCT (Gbagyi, Fulani, Hausa) [29], Nasarawa (Agatu, Basa, Eggon, Hausa) [30] and Niger (Nupe, Gbagyi, Hausa) [31]. Kaduna State in the North-West zone has a majority composed of Gbayi, Hausa and Fulani ethnic groups [32], and Enugu State in South-East is of largely Igbo ethnicity [33]. In terms of religion, Niger (78.6% vs. 17.7%) state has a principally Muslim population (vs. Christian population, respectively), as opposed to FCT (27.4% vs. 68.0%) and Enugu (1.0% vs. 97.3%), which have a principally Christian population [34]. Kaduna (54.2% vs. 45.9%) and Nasarawa (41.4% vs. 56.7%) states have more similar distributions of Muslims versus Christians [34].

### Data collection and organisation

In addition to published protocol and outcomes papers, data sources included Principal Investigators and Study Coordinators of each of the four IR studies. The experiences described highlight major challenges common to study implementation for all the studies, and unique challenges significant enough to warrant mention. Study implementation challenges were thematically categorised under ‘External’ and ‘Internal’ subgroupings. External challenges denote those that were beyond the control of the study teams, namely challenges that needed significant structural, political or other changes in order to be minimised or rectified. Internal challenges are those most amenable to rectification by the study implementation team itself; in other words, challenges easier to control. The details and impact of these challenges are addressed under each theme. For each major challenge encountered, an implemented and/or suggested solution is described.

## Results

Table 1 presents profiles for all four studies, which were conducted in a period spanning 2012 to 2017. Three studies were randomised controlled trials, and one (MoMent) was a prospective cohort study. Each study’s objectives and outcome measures were relevant to at least one of the three 90s of the 90–90–90 initiative. A total of 4431 pregnant women (including 1449 living with HIV) and 1260 HIV-exposed infants were formally enrolled in the four studies. Additionally, the Baby Shower Trial enrolled 2498 male partners. Primary and key secondary outcome results from all four studies have been published, with largely positive findings regarding impact of the interventions tested [18, 19, 21, 22, 24, 35–37].

**Table 1:** Profiles of the four PMTCT implementation research studies in Nigeria, 2012–2017

Profile indicators	Baby Shower	Optimizing PMTCT	MoMent	*Lafiyan Jikin Mata*
Funder	NIH	NIH	WHO/Global Affairs Canada	WHO/Global Affairs Canada
Implementation period	2012–2015	2012–2015	2012–2017	2012–2017
Local PEPFAR implementing partner	Prevention, Education, Treatment, Training and Research-Global Solutions	Friends in Global Health Nigeria	Institute of Human Virology Nigeria	Center for Integrated Health Programs
Study design	Two-arm cluster randomised controlled trial	Two-arm cluster randomised controlled trial	Two-arm prospective matched cohort study	Two-arm cluster randomised controlled trial
Study setting	Enugu state (south east zone)	Niger state (north central zone)	Federal Capital Territory and Nasarawa states (north central zone)	Benue and Kaduna states (north central, north west zones)
Study sites	Catholic/Anglican churches	Primary and secondary healthcare facilities	Primary healthcare facilities	Primary and secondary healthcare facilities
Study settings	Rural and urban	Rural	Rural	Rural and urban
Number of study sites	40 (20 per arm)	12 (6 per arm)	20 (10 per arm)	32 (16 per arm)
Formative studies	2 FGDs with pregnant women, male partners, women’s groups and the clergy	3 FGDs with study participants 10 KIIs with community leaders, clinicians, and local health officials	11 FGDs with pregnant and young women, HIV+ MMs and m2m support group members, male partners 31 KIIs with HIV+ MMs, community leaders, TBAs, HCWs and PMTCT programme implementers and policy-makers	44 FGDs with women including HIV infected pregnant women and their male partners 42 KIIs with HCWs, community women, community and religious leaders, policy-makers
Core interventions	Congregation-based HIV testing for pregnant women	Point-of-care CD4 testing Task shifting Integrated mother-infant service provision Male partner and community engagement	Structured, supervised peer support	Structured continuous quality improvement intervention and breakthrough collaborative series
Control/standard of care	Routine facility-based HIV testing	Routine PMTCT services	Routine PMTCT services including informal peer support	Routine PMTCT services
Study participants	Pregnant women of unknown HIV status Male partners	HIV+ pregnant women HIV-exposed infants	HIV+ pregnant women HIV-exposed infants	HIV + pregnant women
Primary and key secondary outcome measures	Maternal HIV testing during pregnancy Maternal ART uptake HIV testing among male partners of pregnant women	Maternal ART uptake Maternal and infant retention at 6 and 12 weeks postpartum Infant EID uptake by 14 weeks of age MTCT Participant and provider satisfaction Cost-effectiveness	Infant EID presentation by 2 months of age Maternal and infant retention over first 6 and 12 months postpartum Maternal VL suppression at 6 months postpartum Cost-effectiveness	Maternal retention at 6 and 12 months postpartum Infant EID uptake by 10 weeks of age
Original sample size	2700 pregnant women	372 HIV+ pregnant women	480 HIV+ pregnant women	640 HIV+ pregnant women
Revised sample size	N/A	N/A	220 HIV+ pregnant women	520 HIV+ pregnant women
Final enrolment				
Pregnant women	3054	369	497	511
HIV-exposed infants	69	380	408	403
Male partners	2498	N/A	N/A	N/A
Summarised results-main outcomes	Women in IG 11 times more likely to have had an HIV test Male partners in IG 12 times more likely to have had an HIV test	Women in IG 3 times more likely to initiate ART Mother-infant pairs in IG 10 times more likely to be retained in care at 12 weeks postpartum IG infants 74% less likely to acquire HIV infection at 12 weeks postpartum	Infants in IG 4 times more likely to present for timely EID by 2 months of age Mothers in IG 6 times more likely to be retained Mothers in IG 5 times more likely to be virally suppressed	No significant difference in maternal retention at 6 months postpartum Infants in IG 2 times more likely to receive timely EID testing at 4–6 weeks
90–90–90 relevance	First 90 (testing) Second 90 (ART uptake)	Second 90 (ART uptake) Third 90 (viral suppression, presumed due to retention)	Third 90 (viral suppression via adherence) Third 90 (viral suppression, presumed due to retention)	Third 90 (viral suppression, presumed due to retention)

*NIH* National Institutes of Health, *WHO* World Health Organization

*FGD* focus group discussion, *KII* key informant interview, *m2m* mother2mother, *TBA* traditional birth attendant, *HCW* healthcare worker

*EID* early infant diagnosis, *MTCT* Mother-to-child transmission of HIV, *VL* viral load, *N/A* not applicable

*IG* intervention group, *ART* anti-retroviral therapy

### External challenges

#### Healthcare worker (HCW) strikes

Industrial strike actions by organised labour unions are common in Nigeria; these strikes took root in the colonial period of the 1930s to 1950s but persisted through the post-independence 1970s to present times [38, 39]. HCW strikes are unfortunately a notable subset of strike actions in Nigeria, causing shutdowns and lockdowns of healthcare facilities and intimidation by striking HCWs of colleagues who choose to continue to provide services in spite of the strike.

Exposure to, and impact of, HCW strikes were most notable for the INSPIRE studies [40]. During MoMent’s and LJM’s 5-year study period, there was an annual average of three state and/or national-level HCW strikes directly affecting the studies. The duration of the strikes ranged between 5 days and 3 ½ months. The major reason for these HCW strikes was unpaid and/or irregular salary payments, with some employees not being paid for up to 8 months. Ultimately, study implementation and participants’ access to study sites were compromised. LJM sites were exposed to HCW strikes for a median proportion of 18.8% (IQR 0.0–40.0%) of the site-level study implementation period; individual study participants were exposed to HCW strikes for a median of 22.5% (0.0–100.0%) of their study enrolment period. Clearly, retention outcome measures (which are dependent on missed or made participant appointments) were also affected. Invariably, recruitment was up to 50% less efficient, and overall timelines for the INSPIRE studies were significantly delayed, necessitating multiple requests for no-cost extensions from funders.

#### HCW turnover

HCW turnover occurred in two main ways; first, HCWs left their jobs due to better opportunities elsewhere, or were re-posted during often impromptu reshuffles of state and/or local government workforce. The latter practice largely affected state government-owned study sites that were secondary or primary-level facilities, and not federally run tertiary facilities. HCWs at a study facility would be promptly transferred to work at a different facility often in a completely different district; their replacement would come from a different facility and not necessarily have the same level of experience or motivation as the deployed worker. State Ministries of Health engaged in this workforce reshuffling under state and local government practice, a prerequisite for every HCW engaged. The purpose is to ensure that human resources for health are equitably distributed across all districts and sub-districts in local government areas. However, this is counterproductive in study settings, as consistency of study-trained HCWs is a major requirement in protocol compliance and data quality.

Reshuffling of state-employed HCWs occurred once in each study state during LJM and MoMent implementation. For the LJM study, 15 out of 16 study facilities in Kaduna experienced transfer of one or both study nurses in the course of the study. Similarly, at least 40% of the study facilities in Benue experienced transfer of LJM study nurses. This reshuffling practice often led to abrupt disruption of study implementation, especially at intervention sites, where new and/or intensified strategies were being implemented by/with HCWs. Study orientation and training on tools therefore had to be conducted afresh for transferred-in and/or newly hired HCWs.

Clinic staff deployments were not that much of a concern for the Optimizing PMTCT study. Due to prior experience with high staff turnover in the first year of their PEPFAR (President’s Emergency Plan for AIDS Relief) programme, the local implementing partner (Friends in Global Health Nigeria) included a clause in its memorandum of understanding with the Niger state government in subsequent years that required prior consultation before a trained health worker was deployed to a different facility.

#### Acts and threats of ethnic and politically motivated violence and terrorism

Security in Nigeria became an increasing concern after the intensification of Boko Haram’s local activities in 2009. The areas most affected by Boko Haram are in northern Nigeria, specifically in the North-East, North-Central, and North-West. Ultimately, the INSPIRE studies were most affected by threats or acts of Boko Haram terrorism at their study sites. This translated into significant disruptions in the lives of affected HCWs, participants and research staff, in addition to travel restrictions for supervising research staff from Central Offices in the capital city Abuja.

Ethnic violence affecting the studies occurred largely around the border areas and within Nasarawa and Benue States, again impacting the INSPIRE studies specifically. This type of conflict was highly unpredictable. Some site-based study staff had to be transferred to work at other sites due to ethnically related threats of violence. Other staff who stayed in violence-prone areas remained under threat of violence along with their families. One MoMent study site had to be replaced due to constant threat of ethnic violence and extremely poor participant recruitment.

All four studies were affected by restricted movement during presidential and gubernatorial elections affecting the entire country or specific states, respectively, due to fear of, or actual violence or unrest.

#### Multiplicity of local ethical review applications

Ethical review and oversight for research studies is critical for establishing and maintaining adequate protection for study participants. Ethical oversight from local Institutional Review Boards (IRBs) also ensures that studies are implemented in a manner that takes the local context into consideration, including norms, values and special vulnerabilities. This is important for IR, which is expected to be highly context-specific. The Nigerian Code of Health Research Ethics states that a study involving more than three sites should be reviewed by the National Health Research Ethics Committee (NHREC) [41], presumably to reduce the need for approvals from multiple IRBs at the state or study site level. This option is especially important for IR studies, which typically require multiple sites. There is, however, little specific guidance given by NHREC on coordinated ethical approvals between itself and other in-country IRBs. This deficiency affected the four IR studies, since study teams that had applied for and received NHREC approval were still required by some state institutions and healthcare facilities to additionally apply for full ethical review prior to initiating enrolment in their respective jurisdictions. This resulted in a complicated ethical application process that, being largely paper-based, worsened delays in study implementation. The Optimizing PMTCT study sites were all owned by the Niger state government, which did not have a standing IRB, so NHREC approval was considered sufficient. The Niger state government, however, had to provide written approval for the study to proceed.

### Internal challenges

#### Lack of research capacity and experience among study staff

Competent, experienced research staff are invaluable to the quality and timeliness of study implementation and the quality and validity of data generated. Due to the relative lack of large, prospective, well-funded studies and research consortia in Nigeria [12], it is challenging to find well-trained, experienced research staff for IR. As experienced by all four studies, available staff for these positions are often recent graduates and/or health workers looking to change careers or to broaden their research experience. Even so, these individuals’ research experience was limited mostly to short-duration, cross-sectional knowledge–attitude–practice studies. Thus, knowledge and expertise in research administration and study implementation, including application for and renewal of IRB approvals, informed consent, standard operating procedures, research data collection, validation, cleaning and storage, and preparation of financial and progress reports, were quite limited among Nigerian study staff. As such, large prospective studies such as the four described in this paper face constraints in recruiting competent, experienced research staff.

#### Research staff turnover

The turnover of these staff was also high, because once trained and experienced in research and project management, the staff became more attractive to other organisations. Some staff were recruited for more lucrative local jobs, more often in public health than in research, while some succeeded in gaining admission into educational programmes abroad. Additionally, the relative dearth of experienced research staff meant that less competent staff with little or no interest in research were sometimes the only choices for recruitment. Such personnel would leave as higher-paying job opportunities became available, regardless of the learning opportunities with the IR projects. Finally, the relatively sparse research environment provided limited opportunities for continued/stable employment. Therefore, as IR studies neared completion, some staff left prematurely to find more permanent employment opportunities.

#### Travel restrictions resulting from security issues

Study staff were often restricted from travelling to study sites experiencing ongoing violence or threats of violence. During these uncertain times, local staff were able to present to work during peaceful/stable periods, yet centrally located staff in Abuja or in study state capitals were more restricted in travelling to these areas. This situation caused significant interruptions to site supervisory visits provided by centrally located research staff.

## Discussion

### Implemented and suggested solutions

#### Solutions to external challenges

HCW strikes and turnover are difficult to predict and manage at the team level. Studies reporting impact of HCW strikes on the implementation of prospective trials are lacking. A few studies have reported deleterious effects of Nigerian HCW strikes on patient care, including maternal morbidity and mortality [42, 43], and missed appointments for acute and chronic eye care [44]. Given the significant impact of HCW strikes on participants and projects, all four IR teams developed implementation plans with extra time built-in to allow for these occurrences. Furthermore, study teams prepared funder reports that included detailed narratives of these disruptions, in addition to maintaining a calendar to document the strikes and their duration. These detailed records provide resources for taking these disruptions into consideration as confounding factors, if necessary, during statistical analysis.

HCW turnover was planned for and/or managed with careful selection of multiple HCWs, where available, to train on study activities. Training and re-orientations were also planned regularly during the studies in order to accommodate newly posted HCWs at study sites. In the case of the Optimizing PMTCT study, lessons learned from prior work in Niger state enabled the adoption of language in memoranda of understanding with governing authorities that made arbitrary staff transfers less likely. While the development of such memoranda of understanding is helpful, the terms and conditions should be honoured by all parties involved for optimal functionality.

Acts or threats of political/ethnic violence and terrorism were a persistent reality, especially for the INSPIRE studies. As in the case of HCW strikes, contingency plans included developing study timelines with extra time built in to accommodate these disruptions. As mentioned previously, study sites exposed to what was deemed as extreme levels of insecurity were replaced in order to preserve cluster size and maintain collection of data to facilitate robust statistical analysis. However, the safety of study staff was the topmost priority. Study coordinators subscribed to and tracked news outlets and networks for the most up-to-date information on the location and extent of insecurity and violence. All staff in the field were contacted when situations arose that could impact their safety.

The multiplicity of local ethical reviews complicated the ethical approval process and caused delays in study timelines. The need for each IRB and government institution in the study communities to be informed and knowledgeable about the study is rational, yet requiring full review and approval from each study site/state IRB made the process tedious and duplicative. A solution to this challenge lies in fostering the ability of local IRBs to communicate and share information with each other, thereby reducing the burden associated with the ethical approval process while ensuring protection of all participants. Similar challenges with multicentre studies in the United States have led to moves to streamline the ethical review process and strengthen the use of centralised systems [45]. In Nigeria, NHREC continues to play a dominant role, but sub-national IRBs still function quite independently and often do not take into consideration ethical approvals obtained from NHREC or elsewhere.

#### Solutions to internal challenges

One strategy to counter the relative lack of IR capacity among study staff was to establish IR mentoring before and during active implementation of the studies. For example, a team of junior investigators was selected to work closely with the Principal Investigator during grant writing for the Baby Shower trial. Once the study was funded and in active implementation, the junior investigators served as study coordinators, with each assigned to coordinate activities for a specific study aim. The study as a whole is now used to train upcoming junior investigators on various IR skills. Aside from baseline training, the MoMent study held regularly scheduled IR learning sessions called ‘MoMent School’ to address pre-existing and emerging research skill deficiencies noted during study implementation. Additionally, MoMent supported its junior staff to enroll in online and in-person short research courses offered by local and foreign institutions.

Once available in 2014, the WHO IR toolkit [46] served as a useful reference guide, especially for educating new study staff on IR principles. Sections on ‘Introduction and Basic Orientation’ and ‘Planning and Conducting an IR Project’ were especially useful as an introduction for new staff and an adjunct to ongoing field experiences for established staff [46]. Involvement of motivated study staff in writing and presenting abstracts and developing manuscripts has yielded invaluable and sustained capacity-building results. Overall, employment and volunteer opportunities for local staff working on the WHO and NIH studies [12, 47] were in themselves IR capacity-building opportunities.

To protect investments made in the training and re-training of research staff, we suggest making retention of trained, experienced and competent research personnel a priority. Several approaches could be used to achieve this, including having these staff absorbed and retained between studies at their local academic/public health/research institutions. This would make it easier to re-assign them to research projects once a new study becomes active. Prioritised hiring of experienced research staff could also be implemented across institutions, using platforms such as what NISA offers. Such platforms involving multiple governmental, non-governmental and academic institutions can enable individual organisations to communicate the availability of research staff that are completing a study. These staff would therefore be available for engagement on new or ongoing studies seeking qualified staff. These approaches allow for enhancing capacity among an expanding pool of retained research-experienced staff. As an example, the two core research coordinators from Baby Shower were absorbed by the University of Nigeria into their Research Resource Center, subsequently working on a different study in another state [48]. Additionally, five staff from the MoMent and LJM studies were absorbed into NISA’s newly funded Adolescent Coordinated Transition study, currently implemented nationwide [49]. Furthermore, the project coordinator for MoMent was retained after the study and is currently pursuing a PhD under a Fogarty grant targeting Nigerian trainees [50].

Travel restrictions secondary to threats or acts of violence or unrest hindered the implementation and supervisory activities of all four studies, albeit to different extents. The hiring of qualified community members to work at the study sites was extremely useful in minimising turnover and interruptions in site-level study activities, especially at study sites located in unstable areas. The potential travel restrictions for supervising central staff indicate that locally hired staff in these areas would be required to be highly self-driven and proactive since they may receive fewer supervisory and/or mentoring visits.

Ultimately, if studies fail to document details and perform root cause analyses of their challenges, it will be difficult to understand the issues and to develop impactful solutions. For IR projects, consideration of the hybrid models described by Curran et al. [51] or similar approaches for study design is recommended. The three hybrid effectiveness-implementation models described by Curran et al. [51] encompass a priori approaches to testing strategies and/or documenting events relating to clinical effectiveness and/or study implementation. The four studies discussed here were most consistent with the hybrid Type 1 model, where research staff measured effects of study interventions on defined outcomes while observing and gathering information on study implementation. In effect, ‘study implementation diaries’ were maintained alongside data collection for intervention-related outcome measures.

## Conclusions

IR in Nigeria is still in its infancy, and the country continues to face challenges in its quest to contribute to the regional and global body of evidence for HIV and other diseases of public health importance. The major obstacles confronting IR studies in Nigeria include frequent HCW strikes, high turnover of HCWs and research staff, dearth of experienced research staff, insecurity, and burdensome ethical review processes. Nigeria is a priority country in the Global HIV response – its research environment can and has to be improved to facilitate the generation of impactful evidence. Lessons learned from past and future research projects will assist in the development of strategies and policies to foster an enabling environment for IR in Nigeria and similarly challenging settings.

## Abbreviations

ART: Antiretroviral therapy
FCT: Federal Capital Territory
HCW: Healthcare worker
INSPIRE: Integrating and Scaling up PMTCT through Implementation Research
IR: Implementation Research
IRB: institutional review board
LJM: *Lafiyan Jikin Mata*
NHREC: National Health Research Ethics Committee
NIH: National Institutes of Health
NISA: Nigeria Implementation Science Alliance
PMTCT: Prevention of mother-to-child transmission of HIV
